# Providing health sciences education through virtual reality experiences

**DOI:** 10.5195/jmla.2023.1632

**Published:** 2023-10-02

**Authors:** Gail Kouame, Jennifer Davis, Lachelle Smith

**Affiliations:** 1 gmkouame@southalabama.edu, Director, Charles M. Baugh Biomedical Library, University of South Alabama, Mobile, AL.; 2 jdavis14@augusta.edu, Scholarship and Data Librarian, Robert B. Greenblatt, M.D. Library, Augusta University, Augusta, GA.; 3 lsmith411@gatech.edu, Digital Learning Support Specialist, Georgia Institute of Technology, Atlanta, GA.

**Keywords:** Virtual reality, gamification, health sciences library, technology in curriculum

## Abstract

In 2020 – 2021 the Robert B. Greenblatt, M.D. Library at Augusta University implemented two projects leveraging virtual reality (VR) technology to provide immersive experiential learning opportunities for health sciences students. The projects shared some commonalities in spite of having differing objectives and desired outcomes. These common facets led to the success of both projects and will be helpful for other institutions considering implementing VR projects.

One of the projects involved creating a virtual reality room in the Greenblatt Library to provide a readily accessible space for students to engage with the equipment and VR experiences during the library's open hours. This project is described in detail in a previously published article [1] and was also featured in the library's newsletter (https://reeselibrary.wordpress.com/2022/03/11/virtual-dimensions-studio-at-the-greenblatt-library-open-for-business/).

The second project utilized virtual reality technology to create a VR escape room to introduce concepts of data literacy and research data management in a more engaging way. The primary objective of this project was to instruct graduate students in the health sciences disciplines how to ethically manage data. The scholarship and data librarian was inspired to find a way to gamify the information and decided to develop the virtual reality escape room. She and the allied health sciences librarian collaborated with the Department of Physical Therapy (PT) to launch a pilot project to develop the data literacy modules for the PT graduate students. The escape room game is an innovative component to engage students in a non-traditional way beyond the classroom setting. Students work their way through the escape room by finding clues, answering questions and following instructions related to data management principles. Research shows that game-based learning can increase student learning by incorporating elements of game mechanics with established learning theories [2].

Both projects involved partnering with Augusta University's School of Computer and Cyber Sciences, in particular a faculty member who is an expert in virtual and augmented reality. His students chose to build out the VR experiences for the library's projects as capstone projects for their degree programs. This expertise proved invaluable as the library's faculty and staff did not have the skills to build the experiences themselves. The other common elements for both of these projects are that they both involved partnership and collaboration with other academic units on campus (the Medical College of Georgia, the College of Allied Health Sciences, and the School of Computer and Cyber Sciences). They also both utilized the same equipment, allowing for interoperability (see Figure 1). Lastly, they were both funded by awards from the Network of the National Library of Medicine.

**Figure 1:**
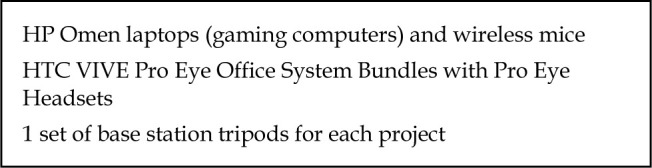
Equipment purchased for VR projects

Both of the projects have been well-received. The virtual reality space in the Greenblatt Library is being utilized by at least three groups per week. The Physical Therapy students provided positive feedback about the virtual reality escape room as a method to learn data management principles.
